# PoseShot: hybrid CNN–BiLSTM transformer model for free throw action recognition via pose analysis

**DOI:** 10.1038/s41598-026-41025-0

**Published:** 2026-03-01

**Authors:** Wei-Chun Hsu, Cheng-Chi Lee, Yong-Hsiang Lee, Yi-Jia Lin, Yi-Chuan Hung, Chien-Chang Ho, Yung-Chun Chang

**Affiliations:** 1 Graduate Institute of Biomedical Engineering, National Taiwan University of Science and Technology, Taipei, Taiwan; 2 Department of Library and Information Science, Fu Jen Catholic University, New Taipei City, Taiwan; 3 Department of Computer Science and Information Engineering, Asia University, Taichung City, Taiwan; 4 Department of Athletics, National Taiwan University of Science and Technology, Taipei City, Taiwan; 5 Graduate Institute of A.I. Cross-disciplinary Technology, National Taiwan University of Science and Technology, Taipei, Taiwan; 6 Sports Administration, Ministry of Education, Taipei, Taiwan; 7 Department of Sport Management, National Taiwan University of Sport, Taipei, Taiwan; 8 Department of Physical Education, Fu Jen Catholic University, New Taipei City, Taiwan; 9 Sports Medicine Center, Fu Jen Catholic Hospital, New Taipei City, Taiwan; 10 Graduate Institute of Data Science, Taipei Medical University, New Taipei City, Taiwan; 11 Clinical Big Data Research Center, Taipei Medical University Hospital, Taipei, Taiwan; 12 Office of Physical Education, Fu Jen Catholic University, New Taipei City, Taiwan; 13 Center for Health Research and Innovation, Fu Jen Catholic University, New Taipei City, Taiwan

**Keywords:** Human action recognition, Multi-channel deep learning, Hybrid transformer, Computer vision, Basketball, Engineering, Mathematics and computing

## Abstract

The evaluation of basketball free throw techniques has traditionally relied on subjective assessments, which introduces inherent biases and inconsistencies in performance analysis. This study presents PoseShot, a novel dual channel hybrid CNN-BiLSTM-Transformer Model that facilitates the comprehensive analysis of free throw mechanics with data-driven insights. Unlike conventional human activity recognition that focuses on coarse activity labels, PoseShot is designed to analyze fine-grained, phase-dependent mechanics within a single basketball free throw motion. The proposed framework integrates training footage with precise body posture angle calculations via a dual-channel deep learning architecture to enable the capture of subtle technical variations. Our innovative approach synthesizes convolutional neural networks (CNN) for spatial feature extraction, bidirectional long short-term memory (BiLSTM) for temporal sequence processing, and a transformer encoder for enhanced contextual understanding of motion dynamics. The model demonstrates exceptional performance, achieving an F_1_-score of 95.76%, precision of 95.72%, and recall of 95.80%. These metrics surpass the performance of established architectures, including DenseNet, Swin Transformer, and Vision Transformer, particularly for the analysis of complex throwing motions. By incorporating both spatial features and postural dynamics, PoseShot provides accuracy in motion analysis. Empirical evaluation reveals PoseShot’s capacity to identify crucial biomechanical determinants of successful free throws, thus offering quantifiable insights for performance enhancement. Since the model’s analysis elucidates the intricate relationship between posture optimization and action consistency, it can provide actionable guidance for athletes and coaches. This research bridges the gap between subjective evaluation methods and advanced motion analytics, establishing PoseShot as a transformative tool in sports performance analysis. The findings demonstrate the potential for data-driven approaches to revolutionize basketball training methodologies through precise, objective assessment criteria.

## Introduction

Great strides in technology have strengthened the connection between Artificial Intelligence (AI) and sports science, particularly in the field of human action recognition (HAR). Thanks to these advances, particularly in machine learning and deep learning, we can now more accurately and efficiently analyze human movements^1,2^. Recent studies have demonstrated AI’s effectiveness in various sports and clinical applications, including gait pattern recognition for biomechanical analysis^3^ and predicting ligament fatigue failure mechanisms to prevent injuries^4^. This not only helps improve sports training but also plays a role in preventing and treating injuries and enhancing overall performance. AI systems, for example, can provide instant analysis and guidance on athletes’ movements to enable faster correction of mistakes and more effective skill development. Alongside HAR, computer vision is becoming a key tool in sports science. By processing and analyzing images and videos, computer vision captures athletes’ body joint positions and offer useful insights for performance analysis. This integration allows sports researchers to obtain deeper insights into the elements influencing athletic performance, which can empower them to develop more efficient training methods and tactics. AI systems, for example, can analyze game footage, identify critical movements, and provide detailed evaluations^5,6^. The continuous advancements in AI and computer vision within sports science afford a wealth of opportunities for enhancing training, optimizing performance, and reducing the risk of injuries. By combining HAR with computer vision, we can gain a deeper, more accurate understanding of human movement in order to provide athletes and coaches with better tools for progress.

According to Statista, basketball is one of the most popular sports globally^7^, with over 400 million regular players who range from amateurs to professionals. This widespread participation underscores basketball’s status as a global phenomenon. Among the many skills in basketball, one of the most crucial techniques is the free-throw shot. Perfecting this skill is vital for players at all levels. However, traditional training methods differ significantly from modern, science-driven approaches that incorporate artificial intelligence. Traditional free-throw training focuses on refining fundamental shooting techniques and body coordination through repetitive practice, such as adjusting wrist, finger, and body movements to achieve consistent, accurate shots. Although effective, this traditional approach often falls short in providing personalized guidance tailored to each player’s specific needs and struggles to accurately measure and analyze throwing performance.

In contrast, AI-powered free-throw training methods prioritize personalization, precise measurement, and real-time feedback. This study leverages AI to deconstruct the fluid motion of free-throw shooting into distinct components, enabling coaches to assess each element in detail. This method facilitates a comprehensive analysis of every aspect of the free-throw, offering valuable insights into the unique characteristics of each movement. With this analysis, personalized guidance and recommendations can be offered to help players improve specific aspects of their shooting technique. By offering detailed, data-driven feedback, this method enhances both technical skills and overall performance. AI-driven training methods also include real-time feedback through sensors and cameras, which allows coaches and players to monitor shooting actions as they happen. This continuous feedback helps players quickly identify and correct bad habits, thus speeding up the improvement process.

Our study offers coaches and players an advanced action classification system. Our model uses a hybrid architecture that combines Convolutional Neural Networks (CNNs)^8^, Bidirectional Long Short-Term Memory (BiLSTM) networks^9^ which is an extension of Long Short-Term Memory (LSTM)^10^, and Transformer layers to process and analyze data. The CNN layers are used to extract features from image inputs, while the LSTM network handles sequential numeric features. Finally, Transformer encoders are applied to further refine these features. By integrating these distinct neural network components, the model enhances both efficiency and speed to provide rapid feedback to coaches and players to improve free-throw technique and overall performance. It is worthy to note that the contribution of this study does not lie in proposing a fundamentally new deep learning architecture. Instead, the novelty of PoseShot stems from its domain-specific formulation for fine-grained basketball free throw action analysis, a problem that has received limited attention in prior HAR research. Unlike generic HAR approaches that focus on coarse activity-level labels, PoseShot explicitly targets the recognition of subtle, phase-dependent shooting mechanics within a single free throw motion. By jointly modeling visual motion patterns and explicit posture dynamics through a dual-channel design, the proposed framework enables objective and biomechanically meaningful analysis of shooting technique that is not achievable with conventional activity recognition pipelines. The main contributions of this work are summarized as follows:

- **Fine-grained action-phase recognition framework**: Unlike conventional HAR approaches that classify coarse activity categories, PoseShot enables phase-level analysis of basketball free throw motions, decomposing a single shooting action into biomechanically meaningful phases (Dribble, Hold, Raise, Throw, Follow-through).
- **Dual-channel posture-vision integration**: We propose a novel dual-input architecture that jointly models visual motion patterns through CNN and explicit body joint dynamics through BiLSTM, enhanced with separate Transformer encoders to capture both spatial features and temporal posture dependencies.
- **Superior performance on fine-grained sports motion analysis**: PoseShot achieves state-of-the-art performance (F₁-score: 95.76%, precision: 95.72%, recall: 95.80%) on basketball free throw action recognition, significantly outperforming established architectures including DenseNet, Swin Transformer, and Vision Transformer.
- **Practical framework for objective performance evaluation**: The system provides data-driven, objective insights into shooting mechanics, enabling coaches and athletes to identify specific biomechanical determinants of successful free throws and receive actionable guidance for technique refinement.

This paper is organized as follows: Sect.  2 provides a foundation for the proposed approach by reviewing existing literature on sports analytics, computer vision, and deep learning techniques used for motion analysis. In Sect.  3, we present the methodology, detailing the application of MediaPipe into the design of a posture-aware system for analyzing free throw techniques along with the deep learning architecture. Section  4 describes our experimental design, including dataset details, the evaluation process, and performance metrics, followed by an in-depth analysis of the results with a focus on accuracy, precision, recall, and other key performance indicators. Section  5 concludes the paper with a summary of key findings, highlights of the study’s contributions, discusses its limitations, and makes recommendations for future research. By tackling the limitations of subjective assessments and incorporating posture-aware analysis, this research provides a valuable contribution to sports analytics and offers practical insights to assist basketball players and coaches in improving free-throw performance.

## Related work

Research on human behavior recognition often relies on image-based inputs, with CNNs serving as a cornerstone in both academic studies and industrial applications. Among the popular CNN architectures, VGG^11^, Inception^12^, and ResNet^13^ each offer unique strengths and advantages. VGG, characterized by its deep architecture of convolutional and pooling layers, is highly effective at extracting detailed features from images. Inception enhances model performance by utilizing its Inception module, which combines convolutional layers of varying kernel sizes within a single layer to capture features at different scales. On the other hand, ResNet incorporates residual learning to address gradient vanishing and exploding issues during training, which enables the development of deeper networks while maintaining high performance. While CNNs have demonstrated strong performance in HAR tasks^14–17^, their computational power is limited compared to Transformer architectures. Known for their ability to capture long-range dependencies and provide a global perspective, Transformers often surpass CNNs in specific applications but encounter difficulties in handling spatial information effectively. By combining CNNs with Transformers, a balanced approach is achieved, where CNNs handle spatial perception and Transformers provide a broader context and thus lead to improved performance in HAR tasks^18^. This combination harnesses the strengths of both architectures by mitigating their individual weaknesses and advancing image recognition from a focus on fine details to a more holistic and comprehensive understanding. While these studies demonstrate strong performance in general HAR tasks, they primarily address coarse-grained activity recognition, rather than the fine-grained, phase-level analysis of a single complex sports motion, such as a basketball free throw.

Another important approach to HAR focuses on extracting joint coordinates from human pose estimation, using these numerical features as input for deep learning tasks. Popular tools for this purpose include MediaPipe^19^ and OpenPose^20^. In our study, we selected MediaPipe for its superior computational speed, which aligns with our goal of achieving higher efficiency. MediaPipe is widely recognized in human pose estimation research for its ability to detect 33 key joint coordinates in a 2D human pose, enabling applications such as live motion analysis, gesture recognition, and virtual reality interactions^21,22^. Its precision in capturing joint coordinates supports a range of applications, from live monitoring systems that track and evaluate user poses to ensure proper posture alignment^23^, to measuring body dimensions for medical and ergonomic uses^24^. With its modular and scalable design, MediaPipe also offers broad applicability in sports, providing instant feedback and tailored analysis, which enhances both the efficiency and accuracy of action recognition tasks^25^.

Building on the strong performance of CNNs and CNN-Transformer models in image-based tasks and numerical data processing for HAR, we aim to achieve a fast and accurate system for recognizing detailed actions by integrating image data with numerical features derived from human joint positions within the images. Therefore, we incorporate the sequential patterns of BiLSTM networks to process the numerical coordinates^26,27^. BiLSTM networks analyze sequential joint movements in both forward and backward directions^28^, providing a more comprehensive understanding of movement dynamics. By combining image and numerical inputs within deep learning models and leveraging BiLSTM’s ability to model dependencies in both directions, we bridge the gap between artificial intelligence and sports science. This fusion has enabled the community to achieve both innovation and high efficiency in action recognition.

## Methodology

**Fig. 1:**
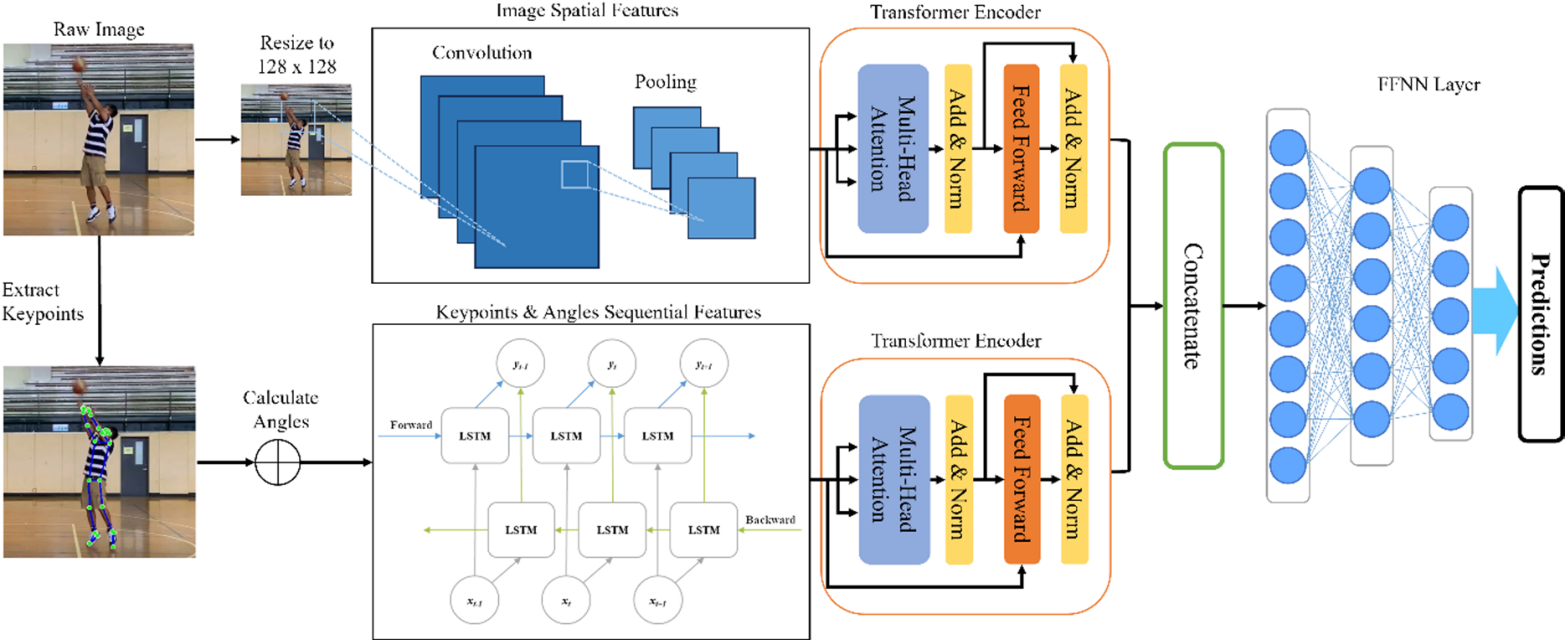
System architecture of the proposed PoseShot pipeline.

This study presents an innovative method for action recognition by combining Convolutional Neural Networks (CNNs) with Bidirectional Long Short-Term Memory (BiLSTM) and Transformer layers to efficiently process both image and numerical data. The goal is to enhance feature extraction from sports videos, with a focus on the classification of basketball movements. The architecture of our proposed method, as shown in Fig. 1, begins with image data being passed through multiple CNN layers, each designed to extract spatial features from the input image. Concurrently, joint coordinates acquired through MediaPipe are processed to calculate key angles associated with joint movements. These joint coordinates are then passed through a multi-layer BiLSTM network, which captures the sequential dependencies of the joint movements.

After extracting spatial and sequential features, the next step involves passing the CNN and BiLSTM outputs into their own Transformer encoder layers. The Transformer plays a crucial role in learning global dependencies within the extracted features, helping to capture long-range relationships across both spatial and sequential information. Using the self-attention mechanism, the Transformer effectively analyzes the entire sequence of features, identifying patterns that are critical for accurate action recognition. This is especially valuable for recognizing complex human actions, where understanding the holistic context is essential for accurate classification. Once the features are processed by the Transformer, the respective outputs are concatenated and passed through a Feedforward Neural Network (FFNN) classifier, which classifies the actions into categories such as ‘Dribble’, ‘Hold’, ‘Raise’, ‘Throw’, and ‘Follow-through’. This architecture, combining CNNs for feature extraction, BiLSTM for sequential dependencies, and Transformers for global context, enables the system to efficiently capture both spatial and sequential dynamics, and this ultimately improves the classification accuracy for basketball action recognition.

### Dataset and preprocessing

**Fig. 2:**
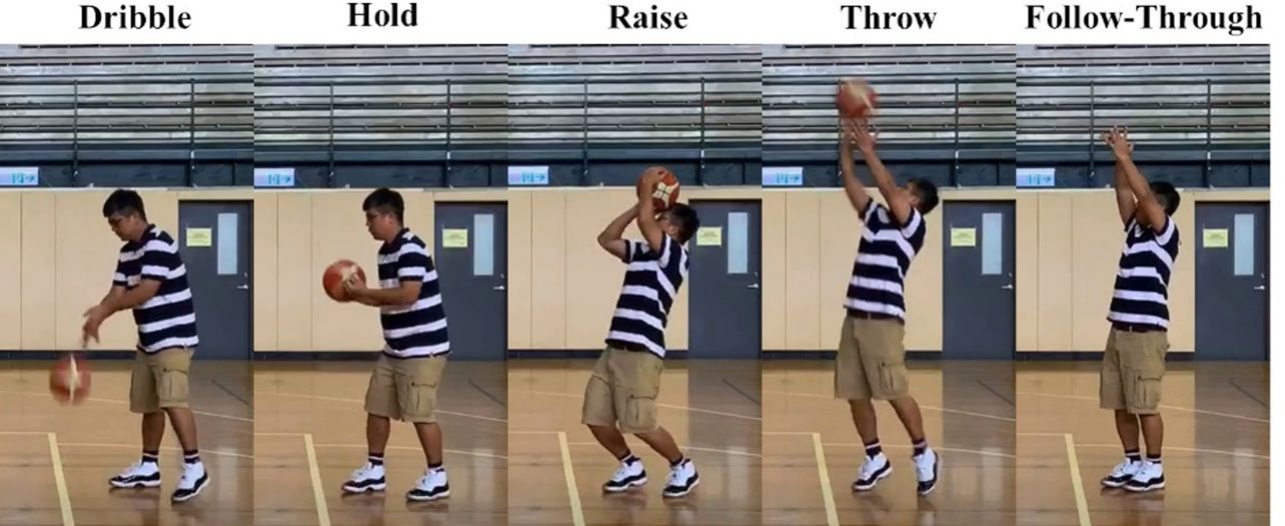
Sample image of each action of a basketball free throw video from the dataset.

**Fig. 3:**
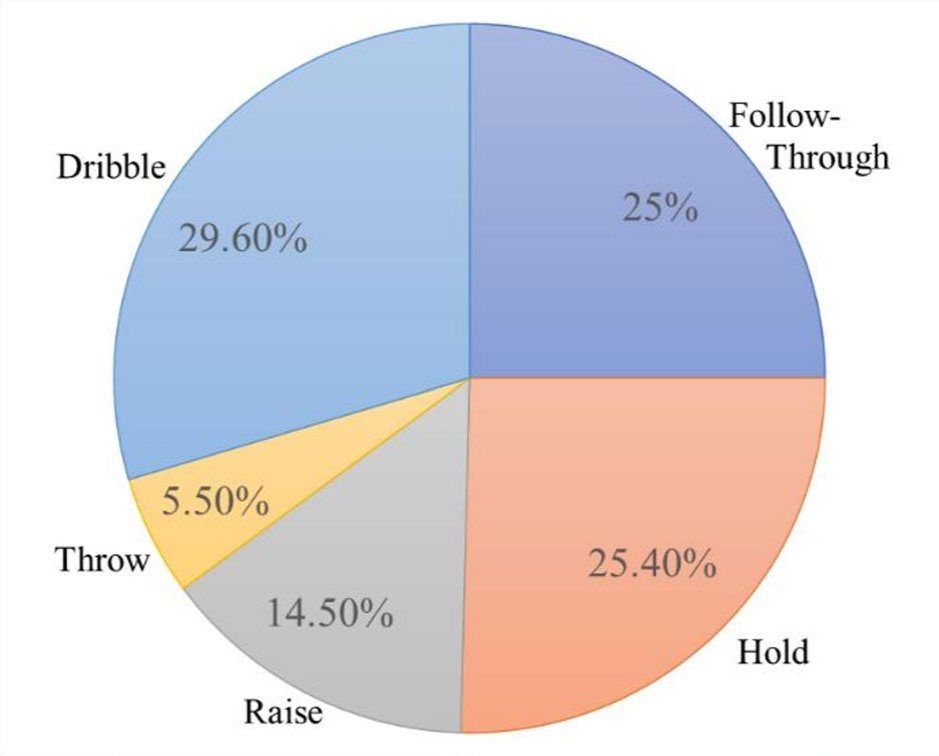
Category distribution in dataset.

To align with the objectives of this study, our dataset is specifically designed for sports action recognition, with a primary focus on basketball shooting actions. The dataset includes a variety of basketball movements, such as dribbling, holding, raising, throwing, and following-through, as shown in (Fig. 2). We sourced this collection of continuous free-throw videos from diverse platforms and annotated them with action labels. In total, 75 videos captured at different frame rates were collected. They have the following distribution of samples per category: Dribble (906), Hold (920), Raise (525), Throw (200), and Follow-through (1072), as detailed in Fig. 3. To ensure comprehensive representation, these actions were captured across a variety of settings, players, shooting angles, lighting conditions, and individual playing styles. This diversity allows our dataset to cover a broad spectrum of action variations, offering a robust foundation for accurate action recognition.

To ensure uniformity and compatibility with our model, each image undergoes two preprocessing steps. First, using the OpenCV library (https://docs.opencv.org/4.x/index.html), we load each original image as a three-channel RGB pixel array. This step standardizes the color representation of the images and allows the model to learn across the channels. After the images are converted to RGB pixel arrays, we resize them to a fixed size of 128 × 128 pixels. Resizing is essential for maintaining uniformity in image dimensions, which is necessary for both training and inference. By standardizing the image sizes, we ensure effective processing and analysis by the model, regardless of their original dimensions. These two preprocessing steps provide consistent input data, which will promote robust and reliable performance across the entire dataset. This approach establishes a strong foundation for subsequent analysis and model training, thus optimizing the dataset’s utility for our task.

### Deriving posture features from raw image data

For extracting pose features from images, we used MediaPipe (https://ai.google.dev/edge/mediapipe/solutions/guide), a powerful tool for joint detection. Our methodology aligned with established practices in HAR research, which commonly enhance coordinate data with additional derived features^29^. Specifically, we derived joint movement angles from the acquired coordinates. Using these key points, we calculated the angles of eight critical joints, including the left and right arms, elbows, hips, and knees, which are expected to yield valuable insights for our study. Examining the sequences of these key points allows us to track fluctuations in joint angles throughout the serve to provide a detailed understanding of the motion mechanics. While MediaPipe provides depth information, our methodology primarily leverages its precise anatomical labeling. Since MediaPipe inherently distinguishes between left and right landmarks with high consistency, this labeled coordinate data provides sufficient spatial context for complex motion analysis without relying on additional depth sensors.

Specifically, we derived joint movement angles from the acquired coordinates. Following the 8-stage kinetic chain model^30^, we calculated the angles of eight critical joints, including the left and right arms, elbows, hips, and knees. These joints represent the essential links in the proximo-distal sequence, where energy is generated from the lower limbs and transferred through the trunk to the upper extremities^31,32^. Examining the sequences of these key points allows us to track fluctuations in joint angles throughout the serve, providing a detailed understanding of the motion mechanics and energy transfer efficiency. As shown in Eq. (1), the angles, represented as$\:{\:A}_{i}$, where $\:i\:\in\:\:[0,\:7],\:i\:\in\:\:N$, correspond to the eight key joints listed in Table 1. In this context, $\:p$ represents the key points (*x*, *y*), while *j*, *k*, and *l* serve as indices corresponding to the respective points listed in Table 1. The angle calculation uses the inverse cosine function along with the mathematical constant π to convert cosine values into angles. These calculated angles are then combined into a feature map, $\:F=[{\:A}_{i},\:{\:A}_{i+1,\:}\dots\:,\:{\:A}_{7}]$, which serves as the input for subsequent model computations. To ensure consistency, the feature map undergoes min-max normalization, as described in Eq. (2). This results in a normalized feature map $\:\widehat{F}=\left[\widehat{{A}_{i},\:\:}\widehat{{A}_{i+1,\:\:}}\dots\:,\:\widehat{{A}_{7}}\right]$, which is then used as the input for our approach. In addition to the joint angles, we included both motion posture coordinates and body joint angles as inputs for the model. These inputs are used to extract latent features for predicting the quality of a series of basketball free-throw actions. Finally, the numerical inputs are represented as $\:V=[\left({x}_{1},{x}_{1}\right),\:\left({x}_{2},{x}_{2}\right),\dots\:,\left({x}_{12},{x}_{12}\right),\widehat{F}]$. The comprehensive procedure for transforming raw visual data into this structured representation is synthesized as in Algorithm 1. This pipeline systematically integrates image-level standardization, including color space conversion and spatial resizing, along with anatomical feature engineering via the MediaPipe framework. This approach effectively encapsulates both spatial positioning and angular kinematics, providing a robust foundation for predicting the quality of basketball free-throw actions.

**Table 1:** Angle and label cross reference.

	Parts (i)	node (j, k, l)
Left	Arm (0)	0, 2, 6
	Elbow (1)	0, 2, 4
	Hip (2)	7, 6, 8
	Knee (3)	6, 8, 10
Right	Arm (4)	1, 3, 7
	Elbow (5)	1, 3, 5
	Hip (6)	6, 7, 9
	Knee (7)	7, 9, 11

1 $$\:{\:A}_{i}={(\mathrm{cos}}^{-1}\left(\frac{{\overrightarrow{p}}_{jk}\cdot\:{\overrightarrow{p}}_{kl}}{\left|{\overrightarrow{p}}_{jk}\right|\cdot\:\left|{\overrightarrow{p}}_{kl}\right|}\right))\cdot\:(\frac{180}{\pi\:})$$

2 $$\:{x}_{scaled}=\frac{x-{x}_{min}}{{x}_{max}-{x}_{min}}$$

**Algorithm 1:**
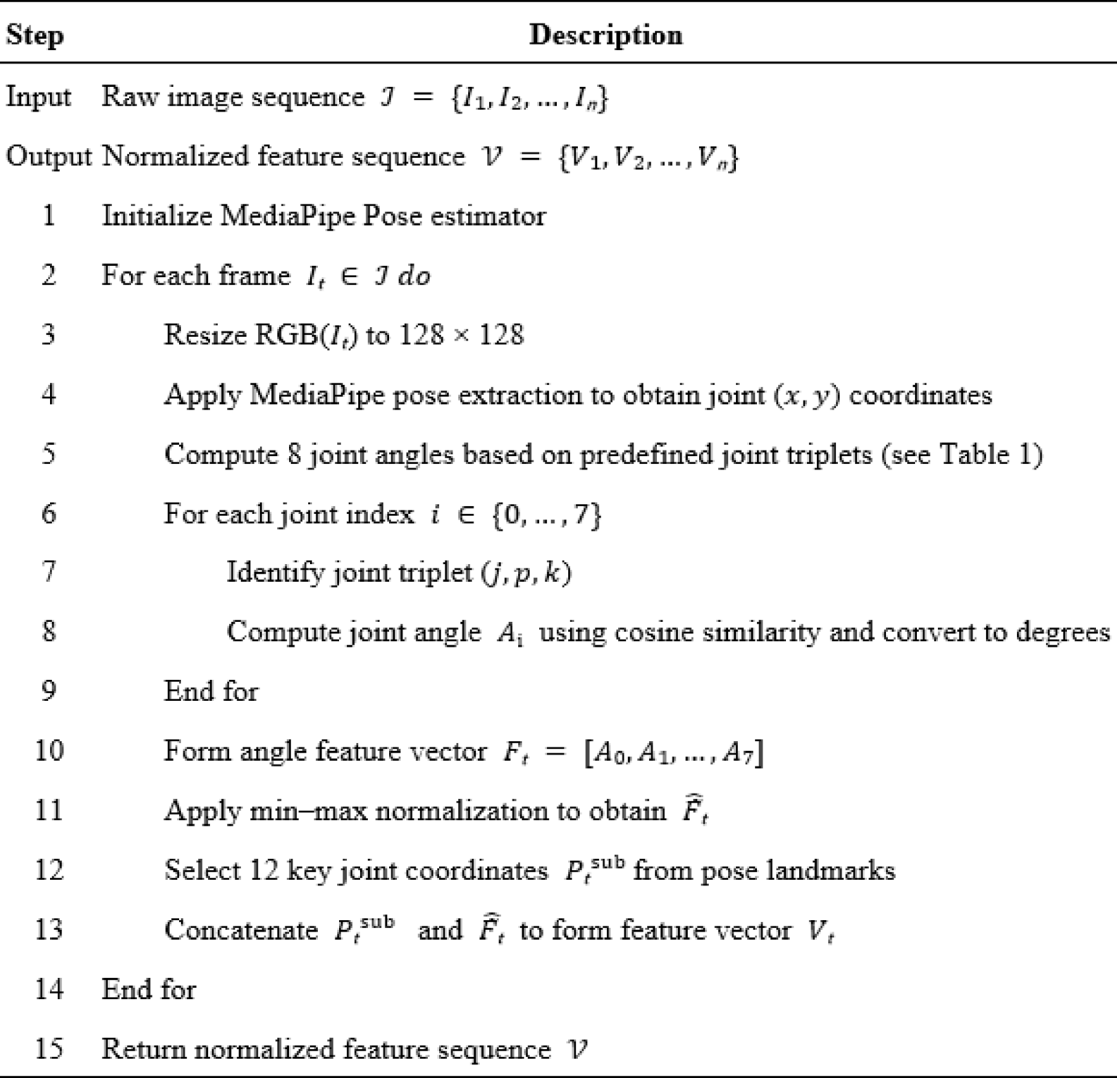
The algorithm of pose feature extraction and normalization.

### The architecture of PoseShot model

This paper presents PoseShot, an innovative hybrid model that integrates two channels: an image channel and a numerical channel. This dual-channel design allows the model to process both sequential and spatial information associated with basketball free throw actions. The main objective is to precisely identify phase-specific actions within the sequence of free throw movements. By combining image and numerical inputs, PoseShot offers a comprehensive perspective, enabling it to effectively capture important features and patterns throughout the entire free throw process. Furthermore, we present an innovative, dual-input, posture-aware framework that leverages the strengths of BiLSTM and CNN. The system extracts image features via CNN layers, which focus on the spatial characteristics of the input images, while also processing joint coordinates through BiLSTM to capture the sequential dependencies in the player’s posture. This dual-input framework is further enhanced by a Transformer encoder layer applied to both the CNN and BiLSTM outputs, allowing the model to analyze both spatial and sequential data more effectively. The outputs of the BiLSTM and CNN channels undergo multi-head attention, which enables the model to focus on important features from both inputs and improve classification accuracy. These refined features are then concatenated into a unified movement pattern representation, combining both spatial and sequential insights. Finally, a multi-class classifier is employed to distinguish specific actions within the free throw sequence, thereby delivering valuable insights for coaching and player improvement.

The following sections provide a detailed overview of the architecture of each layer, highlighting their specific roles within our multi-channel, posture-aware framework for analyzing and predicting actions in basketball free throw motions.

#### Using multi-channel CNN and BiLSTM for extracting underlying features from basketball free throw actions

PoseShot introduces an innovative multi-channel, posture-aware framework that combines BiLSTM and CNN architectures and is enhanced with an attention mechanism. Although the integration of CNN and BiLSTM models has been explored in various fields^33–37^, our work marks a novel application of this methodology to professional basketball sports analysis. To address the diverse nature of the model’s inputs—comprising both image data and numerical sequences of joint angles and key points—customized processing strategies are necessary to manage these distinct data types effectively. As depicted in the model architecture, the system is designed around two primary channels: CNN and BiLSTM networks.

The CNN channel plays a critical role in extracting spatial features from the input images. The architecture of this channel consists of three convolutional layers, each followed by GELU activation, max pooling, and batch normalization. Instead of the more common ReLU activation, we use the Gaussian Error Linear Unit (GELU) activation function as it offers a smoother and more nuanced response to the input data, which makes it particularly effective for handling spatial data. This choice enhances the model’s ability to detect subtle movements, such as those occurring during the free throw action, and as a result, ultimately improves performance and allows the CNN to better capture essential spatial features. The convolutional layers progressively learn more abstract features from the image data as the network deepens. In the initial layers, the filters typically detect simple features, such as edges and textures. As the network deepens, the filters capture more complex patterns and relationships, which are crucial for understanding the intricate structure and dynamics of the image.

- **First convolutional block**: The first convolutional layer extracts low-level features like edges and simple textures. The input image is passed through a convolutional layer with a kernel size of 3 × 3 and 128 output channels. The output size after this operation is reduced due to padding and convolution: For each input image of shape 128 × 128, the output shape becomes 128 × 128 × 128 (height x width x channels). Max pooling is applied afterward, which downsamples the image by half in both dimensions, and results in a shape of 64 × 64 × 128. Batch normalization is then applied to stabilize and speed up training.
- **Second convolutional block**: The second convolutional layer takes the output from the first block (64 × 64 × 128) and further refines the features. This layer uses 32 output channels with a 3 × 3 kernel, which reduces the feature map’s depth while preserving key spatial patterns. After convolution, the output shape is 64 × 64 × 32. Max pooling is again applied to reduce the spatial dimensions to 32 × 32 × 32, followed by batch normalization for stabilization.
- **Third convolutional block**: The third convolutional layer focuses on abstracting the features from the previous blocks. It uses a 3 × 3 kernel with 16 output channels, further compressing the depth while learning more complex spatial relationships. The output shape after convolution is 32 × 32 × 16. Max pooling is applied to reduce the spatial dimensions to 16 × 16 × 16, followed by batch normalization. The output after this block is now 16 × 16 × 16.

After each convolutional operation, max pooling is used to reduce the spatial dimensions of the feature maps, making the model more computationally efficient while retaining the most relevant spatial information. Batch normalization is applied to stabilize the training process and improve convergence. Through these layers, the CNN channel effectively captures progressively more complex spatial features, which is essential for identifying intricate patterns within the input image.

The BiLSTM channel is specifically tailored to capture the sequential dependencies and relationships among joint movements and angles throughout the sequence of actions. In contrast to the CNN channel, which is dedicated to spatial feature extraction, the BiLSTM channel processes numerical data, including joint angles and keypoint sequences, which are extracted from each individual image. This allows the model to understand the dynamic relationships between joints and how they move in relation to each other during the free throw action. The BiLSTM block in PoseShot consists of a 3-layer BiLSTM network. Each layer processes the joint angle data derived from individual frames and captures the dependencies and correlations among these angles across the sequence. The 3-layer BiLSTM structure enables the model to capture both forward and backward dependencies, thus providing a comprehensive understanding of the motion sequence.

#### Enhancing CNN and LSTM representations with transformer encoder layers for free throw analysis

In sports action analysis, integrating multiple neural network architectures has become increasingly prevalent^38–41^, with the aim of extracting complex features and patterns from dynamic data streams. The combination of CNNs and BiLSTM networks has proven exceptionally effective in capturing the spatial and sequential components of sports movements. However, to enhance the ability of these models to represent intricate data, incorporating a Transformer Encoder Layer presents a valuable opportunity to refine features and capture long-range dependencies. Once feature maps are extracted by the CNN block, they are reshaped and passed through a four-layer, four-head Transformer encoder layer. This Transformer refines spatial features by identifying long-range dependencies across the feature map. Following this, torch.max is applied to aggregate the outputs by selecting the most significant features from the transformed data, which are then used as input for the subsequent stages of the model. The application of separate Transformer encoders to process spatial and kinematic feature streams independently has demonstrated effectiveness in action-related recognition tasks^42^. By combining local spatial details with global context, this design offers a more comprehensive understanding of the free throw motion.

Similarly, the BiLSTM output from its final layer is processed through its own four-layer, four-head Transformer encoder. This step enhances the learned sequential features by incorporating long-range dependencies and global context across the joint angles, further refining the model’s ability to analyze and understand free throw motions holistically. The Transformer Encoder layer employs self-attention mechanisms to capture intricate interactions among different parts of the input sequence. This mechanism computes a weighted sum of the input sequence elements based on their significance, where the importance scores are determined by learned attention weights. Let $\:X$ represent the combined output sequence, and $\:{W}_{q},{W}_{k},{W}_{v}$ denote the weight matrices for the query, key, and value projections, respectively. The self-attention mechanism calculates the attention scores, denoted as $\:\mathrm{Attn}\left(X\right)$, as follows:

3 $$\:\mathrm{Attn}\left(X\right)=\mathrm{softmax}\left(\frac{X{W}_{q}{\left(X{W}_{k}\right)}^{T}}{\sqrt{{d}_{k}}}\right)X{W}_{v}$$

where $\:{d}_{k}$ represents the dimensionality of the key vectors. This process allows the Transformer Encoder layer to effectively capture dependencies across different parts of the input sequence. By integrating the Transformer Encoder into our model architecture, we enhance the richness and depth of the embeddings produced by the CNN and BiLSTM networks, which leads to improved accuracy and robustness in our sports action analysis system. Once the attention scores are computed, they are used to weight the elements of the original input sequence $\:X$, which are then combined through a weighted sum operation with a residual connection, thus producing the transformed output sequence:

4 $$\:Transformer\:Output\left(X\right)\mathrm{\:=\:Attn}\left(X\right)+X$$

This operation enables the Transformer Encoder Layer to selectively emphasize various parts of the input sequence and effectively capture the intricate interactions and dependencies within the data. Additionally, the Transformer Encoder facilitates the integration of information across different modalities, such as image data processed by CNNs and sequential data managed by BiLSTMs. This integration harnesses the complementary strengths of each modality and results in more comprehensive and resilient embeddings. The attention mechanism allows the model to capture global dependencies and interactions within the input data, which makes it particularly effective for identifying subtle spatial relationships and long-range sequential dependencies. Incorporating the Transformer Encoder Layer significantly enhances the utility of CNN and BiLSTM embeddings in sports action analysis by enabling the model to grasp complex sequential dynamics and global context within the data. This advancement deepens the understanding of sports movements and improves performance in tasks such as action recognition and prediction, thus driving progress in sports analytics and expanding its applications within the field of computer science.

#### Feed-forward classifier for identifying specific actions in free throw motion

After passing the CNN and BiLSTM embeddings through its respective Transformer Encoder Layer, their outputs are concatenated into a single feature vector. This concatenated vector combines the spatial features extracted by the CNN with the sequential dependencies captured by the BiLSTM in order to provide a comprehensive representation of the free throw motion. The resulting feature vector, which has a combined dimensionality of 512, is then passed through the Feedforward Classifier. The Feedforward Classifier consists of three fully connected layers, each followed by a GELU activation function to introduce non-linearity and improve the model’s ability to learn complex patterns. The first two layers reduce the dimensionality from 512 to 256 and then from 256 to 128, progressively refining the feature representation. The final output of the Feed Forward Classifier is a 5-dimensional vector corresponding to the predicted action categories within the free throw motion. This classification process enables the model to effectively map the concatenated feature vector into the final action categories, which enhances the accuracy and specificity of the predictions.

The AdamW optimizer is used to fine-tune the network’s loss function parameters to optimize model performance. Combined with the cross-entropy loss function, this optimizer promotes efficient convergence and effectively handles sparse gradients, to enhance the stability and robustness of the classification process. For training, we adopted a batch size of 64, set the learning rate to 4e-5, and trained the model over 30 epochs to achieve peak performance. In conclusion, the Feed-Forward Classifier plays a pivotal role in our computational framework by enabling accurate classification of distinct phases within the time-series data of a single free throw. Through the integration of attention-based feature extraction and precise classification, our method demonstrates a powerful synergy of advanced computational techniques, and as a result, effectively addresses the challenges of sports action analysis in the field of computer science.

### Ethics approval

This study was approved by the Taipei Medical University Joint Institutional Review Board (TMU-JIRB Protocol No. N202408036). All methods were performed in accordance with the Declaration of Helsinki and the ethical standards of Taipei Medical University Joint Institutional Review Board. All participants provided written informed consent before participating in video recording sessions for basketball free throw motion analysis. Written informed consent for publication of identifying images in an online open-access publication was obtained from all participants appearing in the figures.

## Experimentation

### Experimental setup and performance metrics

In the experimental setup for this research, the dataset is partitioned into training, validation, and test sets with a split ratio of 7:2:1. This division guarantees that the model is (1) trained on the majority of the data, (2) optimized using a smaller validation set for hyperparameter tuning, and (3) assessed on an independent test set to deliver an unbiased evaluation of its performance. Given the imbalanced nature of our dataset, as illustrated in Fig. 3, we selected F_1_-score as the primary metric for evaluation. The F_1_-score balances both precision and recall, which is particularly useful when evaluating models on imbalanced datasets. While precision focuses on the correctness of the positive predictions and recall on the ability to detect all positive cases, the F_1_-score combines these aspects into a single metric. This makes sure that the model performs well across all classes, particularly the minority classes like ‘Throw’ and ‘Raise’. In addition to the F_1_-score, we also used Precision and Recall as secondary metrics to provide a more comprehensive view of the model’s performance. To further address data imbalance, we employed the macro-average approach, which treats all classes equally by calculating the metric independently for each class and averaging them, regardless of their sample size. This approach ensures that minority classes contribute equally to the overall evaluation. We did not consider accuracy as a primary evaluation metric because it can be misleading in imbalanced datasets. A model that simply predicts the majority classes could still achieve high accuracy, but fail to perform well on the minority classes. By focusing on F_1_-score, precision, and recall, we gain a more detailed and fair evaluation of model performance across all classes.

### Model comparison

To evaluate the performance of our proposed method, PoseShot, we conducted an extensive comparative analysis with several established deep learning models. The objective was to assess PoseShot’s effectiveness relative to state-of-the-art architectures across diverse domains. The comparison included traditional CNNs, known for their proficiency in extracting spatial features, and DenseNet, recognized for its dense connectivity pattern that optimizes parameter efficiency^43^. We also included Inception_v3, praised for capturing multi-scale features effectively^44^, as well as ResNet-18 and ResNet-50, both renowned for their residual learning framework^13^, which facilitates the training of deeper networks. The VGG architecture, widely used in computer vision tasks due to its deep structure and simplicity^11^, was also part of the study. To provide a comprehensive evaluation, we incorporated models based on the Transformer architecture, which excels in handling sequential data and attention mechanisms. Specifically, we compared PoseShot against the Vision Transformer^45^ and Swin Transformer^46^, both offering distinct strategies for image recognition tasks. This diverse range of architectures allowed us to assess PoseShot’s performance across multiple neural network paradigms. Through this comparative study, we aim to showcase PoseShot’s strengths and advantages in the context of deep learning-based systems while gaining valuable insights into its relative performance.

Table 2 provides a comprehensive comparison of various deep learning models for sports motion recognition. Each model demonstrates strengths and weaknesses across the five different actions: ‘Dribble’, ‘Hold’, ‘Raise’, ‘Throw’, and ‘Follow-through’. In this section, we will first highlight the top-performing models for each action, then evaluate the overall performance using key metrics such as F_1_-score, precision and recall. We will conclude by explaining that PoseShot stands out as the top performer due to its ability to effectively balance performance across all actions and its unique hybrid architecture.

**Table 2:** Performance comparison with state-of-the-art methods on our dataset. The best results for each metric are highlighted in bold.

Systems	Pose stage performance (Precision/Recall/F_1_-score in %)					Overall
	Dribble	Hold	Raise	Throw	Follow-through	
Convolutional-based method						
CNN^8^	90.64/94.36/92.46	86.41/90.34/88.33	92.92/88.24/90.52	94.59/74.47/83.33	96.89/96.89/96.89	92.29/88.86/90.31
VGG-16^11^	96.13/89.23/92.55	89.50/92.05/90.76	90.32/94.12/92.18	87.23/87.23/87.23	97.46/**99.48**/98.46	92.13/92.42/92.24
ResNet-18^13^	97.31/92.82/95.01	87.24/**97.16**/91.94	81.20/90.76/85.71	46.15/12.77/20.00	91.58/95.85/93.67	80.70/77.87/77.27
ResNet-50^13^	94.12/82.05/87.67	80.10/91.48/85.41	72.08/93.28/81.32	**100.0**/21.28/35.09	94.36/95.34/94.85	88.13/76.68/76.87
DenseNet^43^	**98.40**/94.36/**96.34**	91.89/96.59/**94.18**	93.33/94.12/93.72	92.50/78.72/85.06	96.97/**99.48**/98.21	94.62/92.65/93.50
Inception_v3^44^	83.51/83.08/83.29	77.17/80.68/79.89	79.20/83.19/81.15	37.50/06.38/10.91	84.47/95.85/89.81	72.37/69.84/68.81
Transformer-based method						
ViT^45^	90.20/94.36/92.23	89.83/90.34/90.08	91.60/91.60/91.60	89.19/70.21/78.57	96.37/96.37/96.37	91.44/88.58/89.77
Swin^46^	96.74/91.28/93.93	90.66/93.75/92.18	92.50/93.28/92.89	85.71/89.36/87.50	98.46/**99.48**/98.97	92.81/93.43/93.09
PoseShot	95.85**/94.87/**95.36	**93.30/**94.89**/**94.08	**95.83/96.64/96.23**	93.62**/93.62/96.17**	**100.0/**98.96**/99.48**	**95.72/95.80/95.76**

Starting with the ‘Dribble’ action, we assess the performance of each model by accurately classifying this action. DenseNet leads with the best F_1_-score of 96.34% and also achieves the highest precision at 98.40%, reflecting its strong ability to make correct positive predictions. PoseShot follows closely with an F_1_-score of 95.36% at second place and stands out by achieving the highest recall at 94.87%; this demonstrates its ability to identify the true positives more effectively than other models, even at the cost of potentially more false positives. ResNet-18 performs reasonably well across all metrics, with F_1_-score, precision, and recall values of 97.31, 92.82, and 95.01%, respectively—this is a solid, balanced performance, particularly in recognizing the ‘Dribble’ action. The models CNN, VGG-16, Vision Transformer, and Swin Transformer perform somewhat similarly, with F_1_-scores ranging from 92.23 to 93.93%; this indicates that while they perform adequately, they still fall short of the top performers in terms of precision and recall. In contrast, Inception_v3 and ResNet-50 perform relatively poorly, with F_1_-scores of 83.29 and 87.67%, respectively. These two models were the only ones to fall below 90% in F_1_-score, which indicates that they struggle more than the other models in accurately classifying the ‘Dribble’ action. These results suggest that despite their deep architectures, Inception_v3 and ResNet-50 may not be as well suited for this specific task, possibly due to the limitations in their ability to capture finer details or handle the nuances in this action.

For the ‘Follow-through’ action, PoseShot leads the way with the best F_1_-score of 99.48% and perfect precision at 100%, indicating its exceptional accuracy in identifying this action without any false positives. Swin Transformer closely follows with an F_1_-score of 98.97% and achieves the best recall at 99.48%, showing its strength in identifying all true instances of the ‘Follow-through’ action, even if it results in slightly more false positives than PoseShot. Notably, both DenseNet and VGG-16’s 99.48% in recall demonstrates their strong performance in detecting the action. However, their F_1_-scores are slightly lower, at 98.21% and 98.46%, respectively, indicating that their precision, while still strong, is not as good as PoseShot’s. Overall, most models perform very well in classifying the ‘Follow-through’ action, with F_1_-scores comfortably above 90%. However, Inception_v3 stands out as the only model that falls short, with an F_1_-score of just 89.81%, which is below the threshold seen in the other models. This highlights that while the ‘Follow-through’ action is generally well recognized, some models struggle to maintain a balance between precision and recall.

Next, we examine the model performance for the ‘Hold’ action. DenseNet takes the lead with the best F_1_-score of 94.18%, alongside a solid precision of 91.89% and a strong recall of 96.59%; this demonstrates a balanced ability to accurately identify the ‘Hold’ action while minimizing both false positives and false negatives. PoseShot follows closely behind, with a difference of just 0.1%, securing second place with an F_1_-score of 94.08%. Yet PoseShot achieves the best precision at 93.30%, highlighting its efficiency in correctly identifying positive instances of the ‘Hold’ action, although slightly trailing DenseNet in recall. ResNet-18 stands out with the highest recall for ‘Hold’, at 97.16%, demonstrating its strength in identifying the true instances of the ‘Hold’ action. However, its relatively lower precision of 87.24% results in a more significant number of false positives, bringing its F_1_-score to 91.94%, which is still decent but not as strong as the top performers. Vision Transformer, Swin Transformer, and VGG-16 all achieve respectable F_1_-scores ranging from 90.08% to 92.18%, showcasing relatively consistent performance across the models. In contrast, Inception_v3, ResNet-50, and CNN showed comparatively poor performance on the ‘Hold’ action, with F_1_-scores of 79.89, 85.41, and 88.33%, respectively. These models were the only ones to fall significantly behind the others and had challenges in classifying the ‘Hold’ action correctly, which might be attributed to overlapping similarities with other actions. For the ‘Raise’ action, PoseShot stands out as the top performer with the highest F_1_-score at 96.23%, along with impressive precision of 96.64% and recall of 95.83%. This demonstrates its robust ability to accurately identify the ‘Raise’ action, excelling in both correctly identifying positive instances and minimizing false negatives. DenseNet follows closely, achieving a strong F_1_-score of 93.73%, along with solid precision at 93.33% and recall at 94.12%, highlighting its reliable performance in classifying this action. Swin Transformer comes next, with an F_1_-score of 92.89% and decent precision and recall values of 92.50 and 93.28%, respectively. This shows that Swin Transformer is effective at identifying the ‘Raise’ action, though it falls slightly behind in overall performance compared to PoseShot and DenseNet. CNN, VGG-16, and Vision Transformer also demonstrate respectable performance, with F_1_-scores ranging from 90.52 to 92.18%, which reflects their reasonable ability to detect the ‘Raise’ action, though they lag behind the top models in precision and recall. In comparison, Inception_v3, ResNet-18, and ResNet-50 performed poorly, with F_1_-scores of 81.15, 85.71, and 81.32%, respectively. These models lagged significantly behind the others and faced greater challenges in correctly identifying the ‘Raise’ action. The relatively low performance of these models suggests limitations in their ability to capture the distinguishing features of this action.

Finally, we examine the ‘Throw’ action, which stands out due to its distinct features in terms of body mechanics, motion, and the interaction with the basketball. PoseShot delivers the best F_1_-score at 96.17%, coupled with the highest recall of 93.62%, along with a precision of 93.62%, demonstrating its exceptional ability to accurately classify the ‘Throw’ action while maintaining a good balance between detecting true positives and avoiding false positives. Swin Transformer comes in second place, but at a distant gap, with an F_1_-score of 87.50%, along with precision and recall values of 85.71 and 89.36%, respectively. While it performs reasonably well, it lags behind PoseShot in both precision and recall, reflecting the challenges it faces in capturing the nuances of the ‘Throw’ action. Other models, including CNN, DenseNet, VGG-16, and Vision Transformer, achieve moderate performance, with F_1_-scores ranging from 78.57 to 87.50%. These models show varying degrees of accuracy in classifying the ‘Throw’ action, but none are able to match the performance of PoseShot in terms of both precision and recall. ResNet-50 achieved the highest precision at 100%, but this came at the cost of a very low recall of 21.28%, resulting in a poor F_1_-score of 35.09%. This suggests that while the model was perfect at correctly identifying the ‘Throw’ action when it predicted it, it failed to capture many true instances, leading to a significant drop in overall performance. ResNet-18 performed even worse, with its F_1_-score of 20% highlighting its difficulty in classifying the ‘Throw’ action accurately. Finally, Inception_v3 really struggled and achieved a very low F_1_-score of 10.91%, demonstrating that the architecture of these models may not be well-suited to the unique features of the ‘Throw’ action, which poses challenges for proper classification.

For overall performance across the five actions, PoseShot leads at the top with the highest F_1_-score, precision, and recall at 95.76, 95.72, and 95.80% respectively. DenseNet and Swin Transformer came in second and third with F_1_-scores of 93.50 and 93.09% respectively. VGG-16, CNN, and Vision Transformer achieves decent overall F_1_-scores ranging from 89.77 to 92.24%. Inception_v3, ResNet-18, and ResNet-50, dragged down by its performance in ‘Throw’ was only able to achieve 68.81, 77.27, and 76.87% in F_1_-scores. PoseShot stands out with its innovative hybrid architecture that seamlessly integrates a CNN for spatial feature extraction, a BiLSTM for processing sequential data, and a Transformer Encoder to optimize the contextual understanding of actions. This unique design enables PoseShot to balance both precision and recall effectively, and thus achieve superior results, particularly in recognizing complex actions like ‘Throw’, where it outperforms Swin Transformer and VGG-16. In conclusion, PoseShot emerges as the top performer in basketball free throw action recognition and surpasses CNN-based and Transformer-based models in precision, recall, and overall F_1_-score. Its combination of attention-based feature extraction and multi-class classification makes it a powerful tool for sports motion analysis, thus positioning it as a promising solution for sports analytics.

## Discussion

**Fig. 4:**
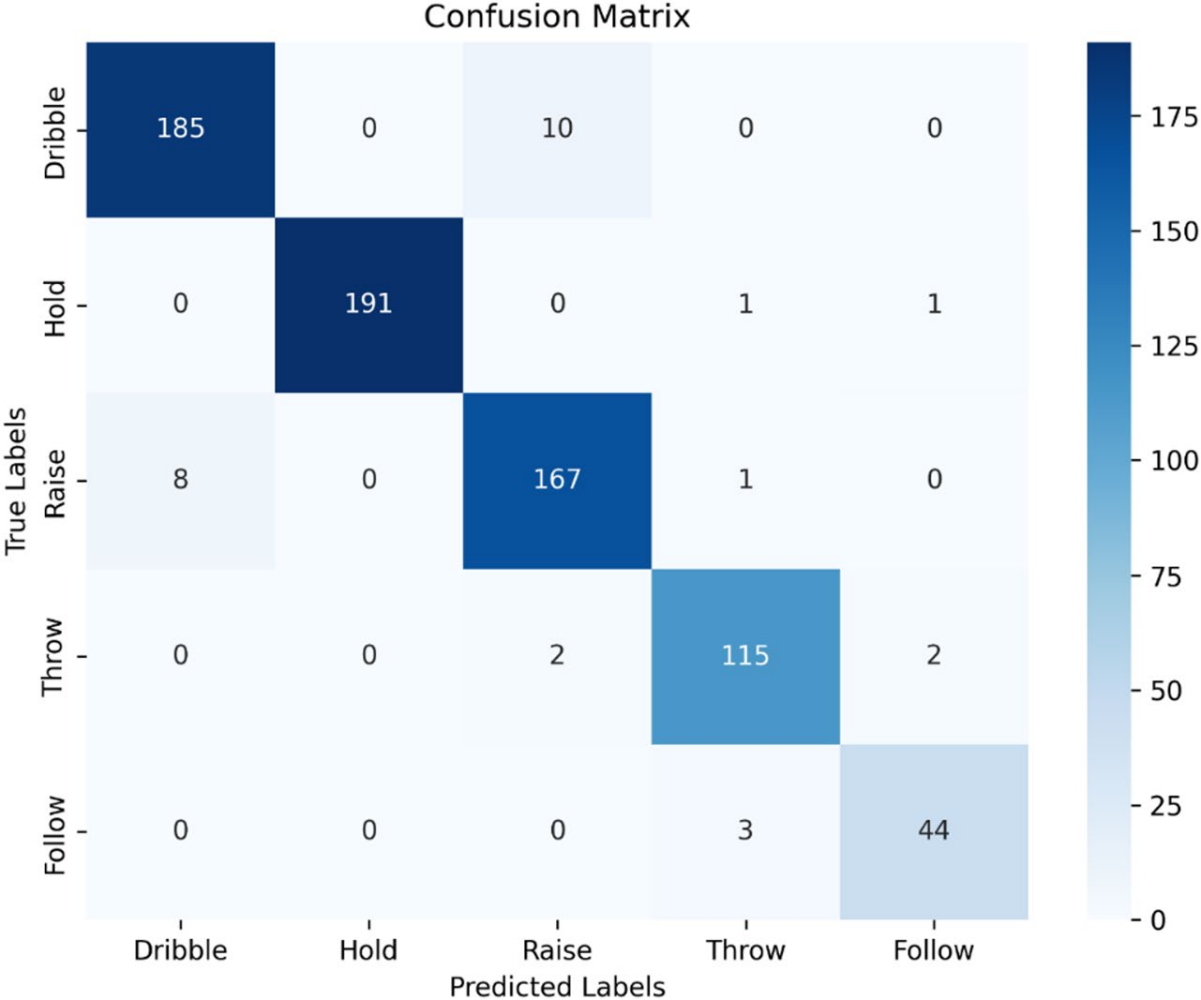
Confusion matrix of PoseShot.

Figure 4 presents the confusion matrix for PoseShot and offers a comprehensive overview of its performance in recognizing the various sports actions of ‘Dribble’, ‘Hold’, ‘Raise’, ‘Throw’, and ‘Follow-through’. Analyzing this matrix provides valuable insights into the model’s strengths and areas for improvement. We will first discuss the ‘Hold’, ‘Throw’, and ‘Follow-through’ actions, and then discuss the actions with the highest number of misclassifications, namely ‘Dribble’ and ‘Raise’. For the ‘Hold’ action, PoseShot performed excellently with 191 correct predictions and only 2 misclassifications, where ‘Hold’ was confused with ‘Throw’ and ‘Dribble’. This highlights the model’s strong ability to recognize actions with distinct spatial features. For the ‘Throw’ action, PoseShot again shows solid performance, correctly classifying 115 out of 119 instances. The 4 misclassifications include 2 instances of ‘Throw’ that were incorrectly predicted as ‘Raise’, and 2 instances that were incorrectly predicted as ‘Follow-through’. These misclassifications are likely due to similarities in arm or body motion during these actions. Further optimization could help PoseShot more effectively differentiate between ‘Throw’, ‘Follow’, and ‘Raise’ to improve accuracy in these instances. The ‘Follow-through’ action had 44 correct classifications, with 3 misclassifications, where ‘Follow-through’ was incorrectly predicted as ‘Throw’. Although PoseShot is effective in recognizing ‘Follow-through’, these misclassifications suggest that there is still some confusion between ‘Follow’ and ‘Throw’. This is likely due to similar movement patterns or posture. Improving the model’s ability to distinguish between these actions would enhance overall classification performance.

When analyzing the ‘Dribble’ action, PoseShot performs well with 185 correct predictions, though there were 10 misclassifications, where ‘Dribble’ was incorrectly predicted as ‘Raise’. While the model excels in recognizing ‘Dribble’, these misclassifications suggest a need for further refinement, especially in distinguishing ‘Dribble’ from actions like ‘Raise’. This brings us to the ‘Raise’ action, which had 167 correct predictions but also 9 misclassifications, where 8 ‘Raise’ actions were confused with ‘Dribble’, and 1 was confused with ‘Throw’. The ambiguities in the dynamic transitions between ‘Dribble’ and ‘Raise’ may account for the misclassifications between these two actions. PoseShot might struggle to distinguish between an ongoing ‘Dribble’ and an initiating ‘Raise’, as their movements can appear similar during these transitional phases, especially when ‘Dribble’ quickly transitions into ‘Raise’ with minimal time spent in the ‘Hold’ phase.

**Fig. 5:**
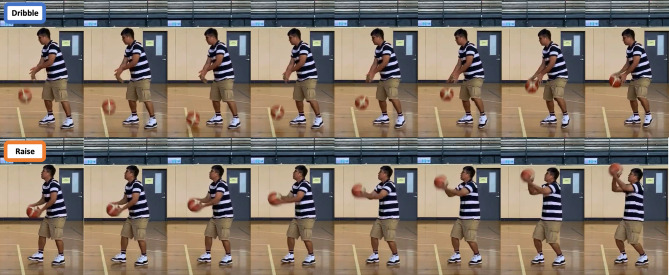
Examples of the ‘Dribble’ and ‘Raise’ poses in a sequence from left to right. ‘Dribble’ can quickly transition to ‘Raise’ without a noticeable ‘Hold’.

Figure 5 illustrates these potential similarities in posture, suggesting that further fine-tuning of the model particularly in the feature extraction process could improve its ability to more effectively differentiate between these two actions. The misclassifications in the ‘Dribble’ and ‘Raise’ categories are particularly noteworthy in the context of basketball free-throw analysis, as accurate identification of these poses is essential to evaluate player performance and technique effectively. To address these issues and improve the model’s accuracy, several strategies can be considered. One potential solution involves incorporating additional features beyond those currently used. For instance, integrating data on body acceleration and hand movements during these actions could provide complementary information to yield a more precise classification. Advanced machine learning techniques, such as ensemble learning and deep feature fusion, may also improve the model’s ability to distinguish between similar poses by combining information from multiple sources. Another approach is the development of specialized training datasets tailored to the nuances of the ‘Dribble’ and ‘Raise’ actions. Curating datasets that contain diverse scenarios and pose variations can help the model learn subtle differences and improving its ability to differentiate these categories. Techniques such as data augmentation and transfer learning can further mitigate challenges posed by limited labeled data, thereby enhancing the model’s capacity to generalize across different conditions.

The difficulty in accurately classifying ‘Dribble’ and ‘Raise’ highlights the complexity inherent in sports motion recognition. This underscores the importance of ongoing research and the exploration of innovative methods to refine recognition systems. By addressing these limitations and implementing new approaches, we can advance the field of sports motion recognition, which will ultimately benefit athlete training, performance evaluation, and sports analytics as a whole. While PoseShot demonstrates strong overall performance, a deeper examination of its limitations reveals fundamental challenges in fine-grained sports motion analysis and specific structural constraints of our current design. The primary limitation lies in distinguishing between ‘Dribble’ and ‘Raise’ actions, which accounted for the majority of misclassifications. This challenge stems from three interconnected root causes. First, these actions share substantial biomechanical overlap, differing primarily in velocity, acceleration profiles, and subtle postural adjustments that may not be fully captured by our 8-joint angle representation. Similar challenges in distinguishing biomechanically similar motion phases have been documented in other fine-grained activity recognition tasks^47^. Second, rapid transitional dynamics create temporal ambiguity: players often transition from dribbling directly into raising with minimal time in a distinct ‘Hold’ position, creating inherently ambiguous boundary frames. Research on action segmentation has shown that such rapid transitions pose significant challenges for temporal models^48^. Third, the current temporal receptive field of our BiLSTM may be insufficient to distinguish between the deceleration pattern of a dribble’s apex and the acceleration initiation of a raising motion, which occur over very short timescales (typically 5–10 frames at standard recording rates). The limitation of recurrent models in capturing such fine-grained temporal dynamics has been noted in prior work on sports motion analysis^49^. Our sensitivity analysis is in Tables 3 and it confirms that while a 3-layer BiLSTM provide the most balanced performance for general free-throw phases, the inherent biomechanical similarity between ‘Dribble’ and ‘Raise’ persists across different parameter scales. This empirical evidence suggests that the confusion is rooted in the action’s fundamental kinematic overlap rather than a lack of model capacity.

**Table 3:** The effect of different layers of Bi-LSTM in PoseShot.

Layers	Pose stage performance (precision/recall/F_1_-score in %)					Overall
	Dribble	Hold	Raise	Throw	Follow-through	
1	95.34/94.36/94.85	91.16/93.75/92.44	95.73/94.12/94.92	93.48/91.49/92.47	99.48/**99.48**/**99.48**	95.04/94.64/94.83
2	**97.30**/92.31/94.74	91.30/**95.45**/93.33	94.31/**97.48**/95.87	93.18/87.23/90.11	97.94/98.45/98.19	94.81/94.18/94.45
3	95.85/**94.87**/**95.36**	**93.30**/94.89/**94.08**	**95.83**/96.64/**96.23**	**93.62**/**93.62**/**96.17**	**100.0**/98.96/**99.48**	**95.72**/**95.80**/**95.76**

A structural limitation is that visual features (CNN) and kinematic features (BiLSTM) are processed independently until final concatenation. This design prevents early-stage cross-modal interactions that could leverage complementary information^50^. For instance, when joint angle sequences are ambiguous (e.g., similar elbow angles during late-stage dribble vs. early-stage raise), visual context such as ball position could provide disambiguating information. However, our architecture only fuses modalities after separate Transformer encoding, limiting cross-modal reasoning capability. Table 4 shows our component-wise ablation study supports this dual-channel necessity: removing either the CNN-based visual stream (M3) or the BiLSTM-based kinematic stream (M2) led to a notable decline in performance. These results empirically demonstrate that while processed independently, both spatial context and skeletal dynamics provide indispensable information that the other modality cannot fully compensate for. This represents a fundamental tradeoff between architectural simplicity and cross-modal interaction, a tension well-documented in multimodal learning literature^51^. The model’s sensitivity to recording condition variations (lighting, camera angles, partial occlusions) reflects deeper structural dependencies. Our CNN pathway is vulnerable to domain shift when deployment conditions differ from training data characteristics^52^. Similarly, MediaPipe’s pose estimation quality degrades under poor lighting or unusual viewing angles^53^, introducing noise that propagates through subsequent layers. These dependencies are compounded by our relatively limited dataset size (3,623 labeled frames across 75 videos). While sufficient for proof-of-concept, this scale limits exposure to the full variability of player biomechanics, shooting styles, and environmental conditions in real-world scenarios. The resulting overfitting risk, though partially mitigated by our train-validation-test split, reflects a gap between architectural complexity and available training samples, a common challenge in specialized sports analytics applications^54^.

**Table 4:** The effect of different component combination in PoseShot.

Components	Pose stage performance (precision/recall/F_1_-score in %)					Overall
	Dribble	Hold	Raise	Throw	Follow-through	
CNN+BiLSTM(M1)	**96.09**/88.21/91.98	84.18/93.75/88.71	93.21/90.76/91.53	89.13/87.23/88.17	98.44/97.93/98.18	92.03/91.57/91.71
CNN+Transformer(M2)	**96.09**/88.21/91.98	84.18/93.75/88.71	92.31/90.76/91.53	89.13/87.23/88.17	98.44/97.93/98.18	92.03/91.57/91.71
BiLSTM+Transformer(M3)	86.42/71.79/78.43	72.04/86.36/75.55	91.51/81.51/86.22	**95.43**/**97.41**/**96.41**	95.43/97.41/96.41	85.75/86.57/85.75
PoseShot	95.85/**94.87**/**95.36**	**93.30**/**94.89**/**94.08**	**95.83**/**96.64**/**96.23**	93.62/93.62/96.17	**100.0**/**98.96**/**99.48**	**95.72**/**95.80**/**95.76**

The multi-layer Transformer encoders (4 layers, 4 heads per channel) substantially increase computational costs compared to simpler baselines. Transformers’ self-attention mechanisms are crucial for capturing long-range dependencies in both spatial and temporal features^55^, enabling holistic motion pattern understanding. Our ablation studies on encoder depth and attention heads confirm that a 4-layer, 4-head configuration provides the optimal balance between feature refinement and recognition accuracy (see Tables 5 and 6). We observed that increasing these parameters further does not yield additional performance gains, suggesting a saturation point in model capacity for this specific action-phase task. This evidence justifies our architectural choices as a strategic trade-off between model complexity and classification precision. However, this increases inference time and memory requirements, potentially limiting real-time deployment in resource-constrained environments such as mobile coaching applications. The computational burden grows quadratically with sequence length^56^, making the approach less scalable to longer video segments or higher frame rates without architectural modifications. These limitations highlight that fine-grained sports action recognition faces inherent challenges beyond conventional activity classification, particularly in handling rapid transitional dynamics and biomechanical ambiguity. Future work should address these root causes through architectural innovations (e.g., cross-modal attention mechanisms), expanded training data, and computational optimizations to balance performance with practical deployability.

**Table 5:** The effect of different transformer encoders in PoseShot.

Encoder	Pose stage performance (precision/recall/F_1_-score in %)					Overall
	Dribble	Hold	Raise	Throw	Follow-through	
2	96.32/93.85/95.06	91.26/94.89/93.04	94.92/94.12/94.51	90.48/80.85/85.39	97.46/**99.48**/98.46	94.09/92.64/93.29
4	95.85/**94.87**/**95.36**	**93.30**/94.89/**94.08**	**95.83**/**96.64**/**96.23**	**93.62**/**93.62**/**96.17**	**100.0**/98.96/**99.48**	**95.72**/**95.80**/**95.76**
6	**96.79**/91.79/94.21	90.81/**95.45**/93.07	**95.83**/**96.94**/**96.23**	89.36/89.36/89.36	98.96/98.96/98.96	94.35/94.44/94.37

**Table 6:** The effect of different attention heads in PoseShot.

Encoder	Pose stage performance (precision/recall/F_1_-score in %)					Overall
	Dribble	Hold	Raise	Throw	Follow-through	
2	97.75/89.23/93.30	86.73/96.59/91.40	95.76/**94.96**/95.36	**95.45**/89.36/92.31	98.97/**99.48**/99.22	94.93/93.92/94.32
4	95.85/**94.87**/**95.36**	**93.30**/94.89/**94.08**	**95.83**/96.64/**96.23**	93.62/**93.62**/**96.17**	**100.0**/98.96/**99.48**	**95.72/95.80**/**95.76**
8	**98.32**/90.26/94.12	89.06/**97.16**/92.93	95.00/95.80/95.40	93.48/91.49/92.47	99.48/**99.48**/**99.48**	95.07/94.84/94.88

In summary, PoseShot performs well in recognizing most actions but struggles with distinguishing between actions that share similar movement patterns, especially ‘Dribble’ and ‘Raise’. The hybrid architecture combining CNN for spatial feature extraction, BiLSTM for sequential data processing, and a Transformer Encoder for contextual refinement proves effective in understanding intricate sports movements. However, practical deployment in real-world basketball training environments requires addressing several considerations beyond performance metrics. Our current implementation processes frames at approximately 15–20 FPS on standard GPUs, which is sufficient for post-training analysis but may introduce latency for real-time feedback during active shooting sessions^6^. The model was trained on videos captured under controlled conditions, and robustness under diverse real-world facilities with varying lighting quality, background complexity, and camera angles remains partially validated. Preliminary observations suggest reasonable performance under moderate variations, but significant deviations in camera positioning or lighting conditions may degrade accuracy, similar to challenges documented in other sports vision systems^57^. Scalability to multi-player scenarios, different skill levels, and integration with existing coaching workflows represent additional deployment challenges. While our dataset includes players across skill ranges, systematic evaluation from youth to professional levels and adaptation to team practice settings require further investigation^58^. These practical considerations highlight that transitioning from controlled experimental validation to operational deployment in diverse basketball training environments necessitates continued refinement in both model robustness and system integration.

## Conclusions and implications

This study introduced PoseShot, a sophisticated Dual Channel Hybrid CNN-BiLSTM-Transformer model that revolutionizes the analysis of basketball free throw actions using advanced computer vision techniques. While traditional assessments rely heavily on subjective observations, which make it difficult to capture subtle technical nuances, our proposed framework demonstrates an exceptional capability in providing objective, data-driven insights. The integration of CNN for spatial feature extraction, BiLSTM for sequential processing, and Transformer Encoder for contextual understanding enables PoseShot to achieve remarkable performance metrics, with an F_1_-score of 95.76%, precision of 95.72%, and recall of 95.80%. Our experimental results reveal both the strengths and limitations of the model. PoseShot exhibits excellent performance in recognizing distinct actions such as ‘Hold’, ‘Throw’, and ‘Follow’, while facing challenges in distinguishing between actions with similar movement patterns, particularly ‘Dribble’ and ‘Raise’. These findings highlight the complexity of sports motion recognition and suggest directions for future improvements, including the incorporation of additional movement features and specialized training datasets.

While PoseShot demonstrates strong performance in fine-grained free throw action recognition, several promising directions remain for future investigation. First, incorporating temporal-frequency features could further enhance the model’s discriminative capability, particularly for distinguishing actions with similar spatial characteristics. Recent advances in temporal-frequency attention mechanisms, such as Fourier attention^59^ and wavelet attention^60,61^, have shown effectiveness in capturing critical patterns within high-dimensional sequential data^62^. These techniques could be particularly valuable for extracting subtle motion patterns in basketball actions, such as wrist flick dynamics or body sway frequencies^63,64^, which may help differentiate between confusable action pairs like ‘Dribble’ and ‘Raise’. Additionally, exploring cross-modal attention mechanisms that enable stronger interaction between visual and pose channels during feature extraction, rather than only at the fusion stage, could yield more robust representations. Finally, extending the framework to analyze other basketball skills (e.g., layups, three-point shots) or adapting it to different sports disciplines would demonstrate its generalizability and practical value across broader sports analytics applications.

Despite these challenges, PoseShot represents a significant advancement in sports performance analysis. Its dual-channel architecture, combining training footage with precise body posture calculations, provides insights into the biomechanical factors contributing to successful free throws. This comprehensive approach not only enhances our understanding of proper shooting mechanics but also offers practical, actionable guidance for players and coaches. As we continue to refine the model and address its current limitations, PoseShot has the potential to transform basketball training methodologies by bridging the gap between traditional subjective assessments and cutting-edge motion analytics. This research demonstrates the possibility of integrating artificial intelligence with sports science to pave the way for more precise, objective, and effective training approaches in basketball and potentially other sports disciplines.

## Data Availability

The datasets generated and/or analyzed during this study are available from the corresponding author upon reasonable request.
